# *Aristolochia yachangensis*, a new species of Aristolochiaceae from limestone areas in Guangxi, China

**DOI:** 10.3897/phytokeys.153.52796

**Published:** 2020-07-16

**Authors:** Ya Jin Luo, Shi Dong Ni, Qiang Jiang, Bo Gao Huang, Yan Liu, Yu Song Huang

**Affiliations:** 1 Management Center of Yachang Orchid National Nature Reserve, Baise, Guangxi, 533209, China Management Center of Yachang Orchid National Nature Reserve Baise China; 2 Guangxi Key Laboratory of Functional Phytochemicals Research and Utilization, Guangxi Institute of Botany, Guangxi Zhuang Autonomous Region and the Chinese Academy of Sciences, Guilin, Guangxi, 541006, China Guangxi Institute of Botany Guilin China

**Keywords:** *
Aristolochia
*, limestone flora, new taxa, north-western Guangxi, taxonomy

## Abstract

*Aristolochia yachangensis* B.G.Huang, Yan Liu & Y.S.Huang, a new species from limestone areas in Guangxi, China, is described and illustrated. It is morphologically most similar to *A. fangchi* Y.C.Wu ex L.D.Chow & S.M.Hwang, *A. petelotii* O.C. Schmidt and *A. championii* Merr. & Chun in shape of leaf blade, anther, gynostemium and inflorescence on old woody stems. However, it can be easily distinguished from the latter by shape of inflorescence, length of upper and lower portions of perianth tube, colour of the limb and throat. A table and a key to distinguish the new species from other morphologically similar *Aristolochia* species are also provided.

## Introduction

The genus *Aristolochia* L. (s. l.) contains 600 species and widely distributes in tropical, subtropical and temperate regions of the world (González 2012; Zhu et al. 2019c). Based on recent studies on molecular phylogeny, chromosome and morphology of *Aristolochia*, some researchers have suggested that an old genus *Isotrema* Raf. should be reinstated to accommodate species of *Endodeca* Raf. and Aristolochia subgen. Siphisia (Duch.) O.C.Schmidt (Zhu et al. 2019a). However, many researchers still advise to use the name *Aristolochia* rather than *Isotrema* (Do et al. 2019; Peng et al. 2019; Cai et al. 2020). In this paper, we use the name *Aristolochia* to describe a new species, because the genus name *Isotrema* is still controversial.

Currently, there are more than 70 species of *Aristolochia* known from China, including many new species that have been described from Yunnan, Guangxi, Guangdong, Zhejiang and Hainan in recent years (Gong et al. 2018; Zhu et al. 2018, 2019b, 2019d; Li et al. 2019; Peng et al. 2019; Zhou et al. 2019). As one of the most biodiverse regions of China, Guangxi has 22 *Aristolochia* species (Peng et al. 2019; Zhu et al. 2019c), including *A. bambusifolia* C.F.Liang ex H.Q.Wen, *A. longlinensis* Yan Liu & L.Wu and *A. gongchengensis*,Y.S.Huang, Y.D.Peng & C.R.Lin, which are endemic in the region (Qin and Liu 2010; Huang et al. 2015; Wu et al. 2015)

During a fieldwork in Yachang Orchid National Nature Reserve of north-western Guangxi, China in April 2019, we discovered a special flowering plant of Aristolochiaceae and speculated that it might be a new species of *Aristolochia*, based on its flower structure. We investigated this species at the same location again and collected specimens of young capsules in May 2019. In order to obtain more detailed morphological data, we came back to the same location once again and collected specimens of mature capsules in July 2019. After consulting Flora of China (Hwang et al. 2003) and other relevant literature (Merrill and Chun 1940; Liang 1975; Chow and Huang 1975; Hwang 1981; Cheng et al. 1988; Ma 1989a, 1989b; Ma and Cheng 1989; Wen 1992; Liu and Deng 2009; Xu et al. 2011; Huang et al. 2013, 2015; Wu et al. 2013, 2015; Do et al. 2014, 2015a, 2015b, 2016, 2017, 2019; Huong et al. 2014; Zhu et al. 2015, 2016, 2017a, 2017b, 2018, 2019b, 2019d; Do and Nghiem 2017; Gong et al. 2018; Li et al. 2019; Peng et al. 2019; Zhou et al. 2019; Cai et al. 2020), as well as comparisons amongst this unknown species and its morphologically most similar species, we confirmed that this species was clearly different from the known *Aristolochia* species. Hence, it is here described and illustrated as a new species.

## Material and methods

Field observations have been conducted in flowering and fruiting *phases* at the type locality more than once. Measurements and assessments of morphological characters of the new species were based on living plants in the wild and specimens gathered from the type locality. All specimens were deposited in the herbarium of Guangxi Institute of Botany (IBK), as well as the herbarium of Guangxi Botanical Garden of Medicinal Plants (GXMG). The comparisons amongst *Aristolochia yachengensis* B.G. Huang, Yan Liu & Y.S.Huang, *A. fangchi* Y.C.Wu ex L.D.Chow & S.M.Hwang, *A. petelotii* O.C.Schmidt and *A. championii* Merr. & Chun were based on the descriptions from herbarium specimens (including types) at CDBI, CSH, CZH, GXMG, GXMI, GZAC, GZTM, HEAC, HITBC, IBK, IBSC, K, KUN, NAS, PE, PEM, SM and protologues (Schmidt 1933; Merrill and Chun 1940; Liang 1975). Images of type specimens and dried herbarium specimens were gathered from JSTOR Global Plants (http://plants.jstor.org), Chinese Virtual Herbarium Website (http://www.cvh.ac.cn/) and Sharing Platform of IBK (http://www.gxib.cn/spIBK/). The materials about current habitat status and threatened factors were recorded in field observations. The assessment of risk of extinction of the new species was based on the IUCN Red List of Threatened Species Categories and Criteria and Guidelines for Using the IUCN Red List Categories and Criteria (IUCN 2001; IUCN Standards and Petitions Committee 2019).

## Taxonomic treatment

### Aristolochia yachangensis

Taxon classificationPlantaePiperalesAristolochiaceae

B.G.Huang, Yan Liu & Y.S.Huang sp. nov.

1F1F5F44-3524-5EBC-9DCD-7D3517F90126

urn:lsid:ipni.org:names:77210596-1

**Figure 1.**
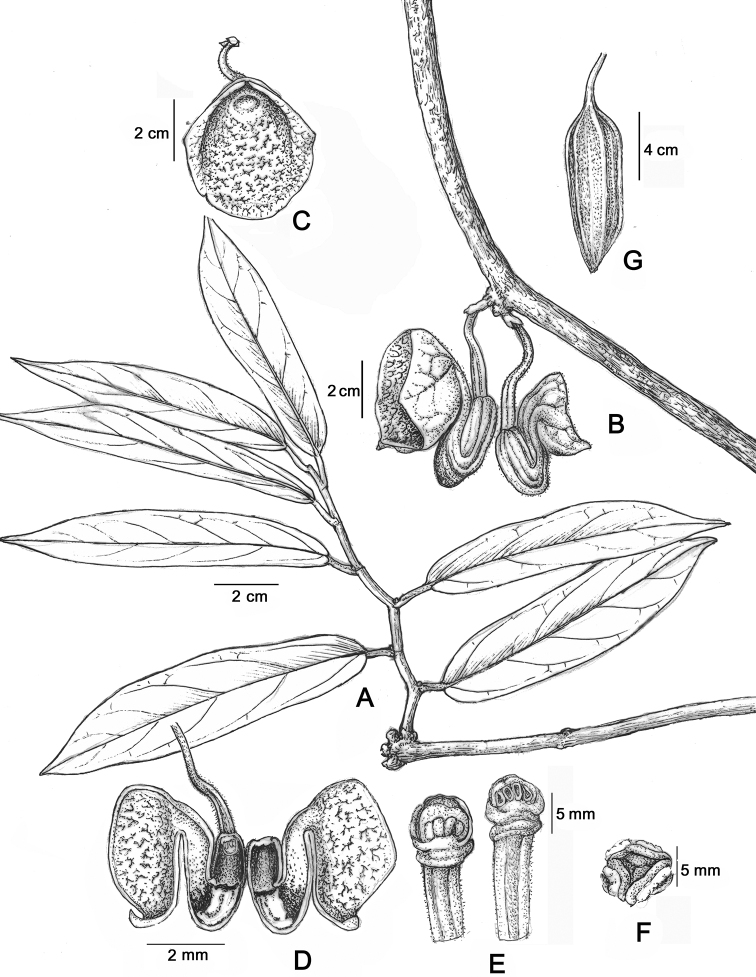
*Aristolochia yachangensis* B.G.Huang, Yan Liu & Y.S.Huang, sp. nov. **A** habit **B** flowering branch **C** flower (front view) **D** longitudinally dissected flower (showing the inside structure) **E** anthers and gynostemium (lateral view) **F** old phase of gynostemium (vertical view) **G** capsule. Drawn by Wenhong Lin (IBK).

**Figure 2.**
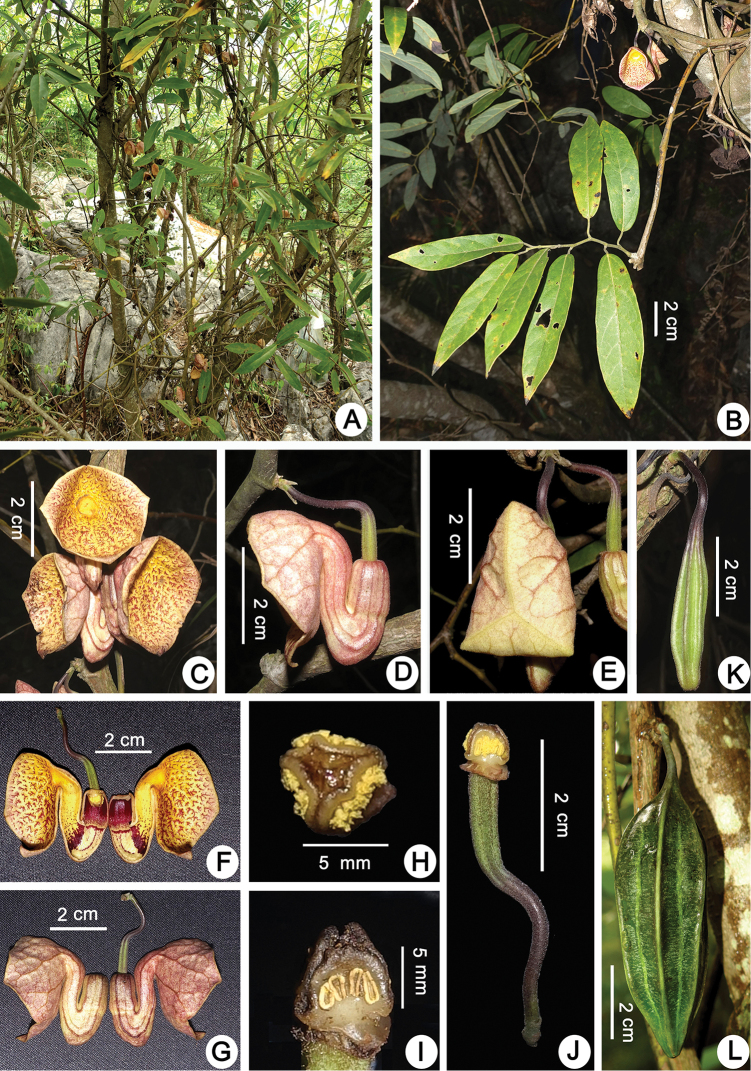
*Aristolochia yachangensis* B.G.Huang, Yan Liu & Y.S.Huang, sp. nov. **A** habitat **B** flowering branch **C** flowers (front view) **D** flower (lateral view) **E** flower bud **F** longitudinally dissected flower (showing the inside structure) **G** longitudinally dissected flower (dorsal view) **H** old phase of gynostemium (vertical view) **I** old phase of anthers and gynostemium (lateral view) **J** ovary **K** young capsule **L** mature capsule. Photographed by Shuwan Li.

**Figure 3.**
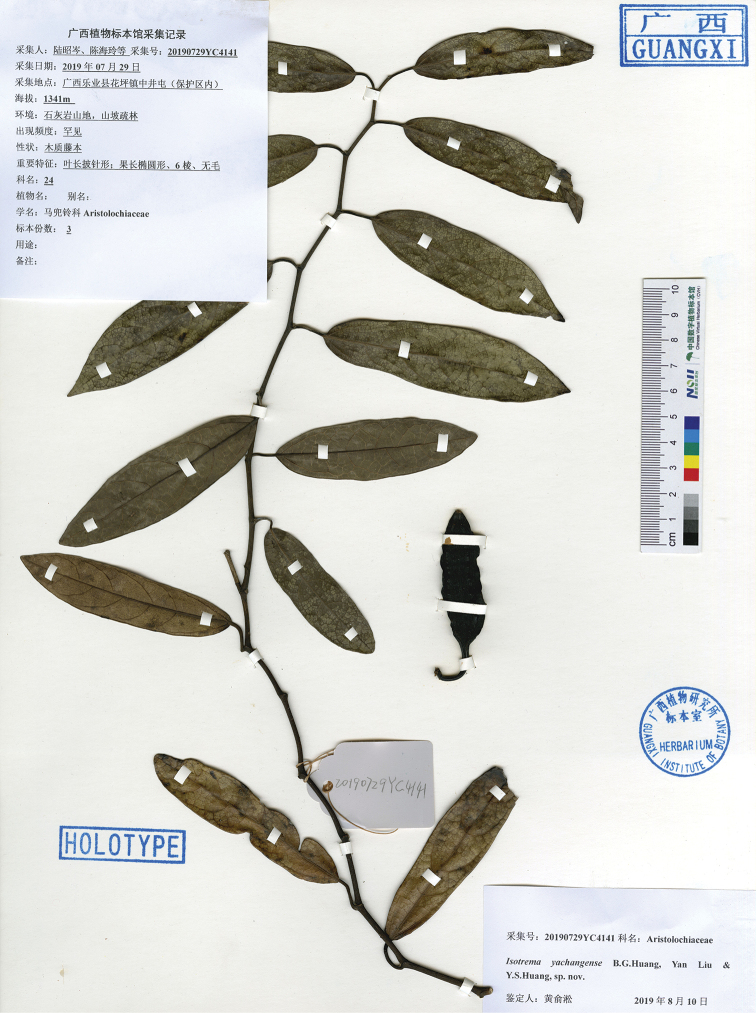
Holotype of *Aristolochia yachangensis* B.G.Huang, Yan Liu & Y.S.Huang, sp. nov. Z. C. Lu et al. 20190729YC4141(IBK).

**Figure 4.**
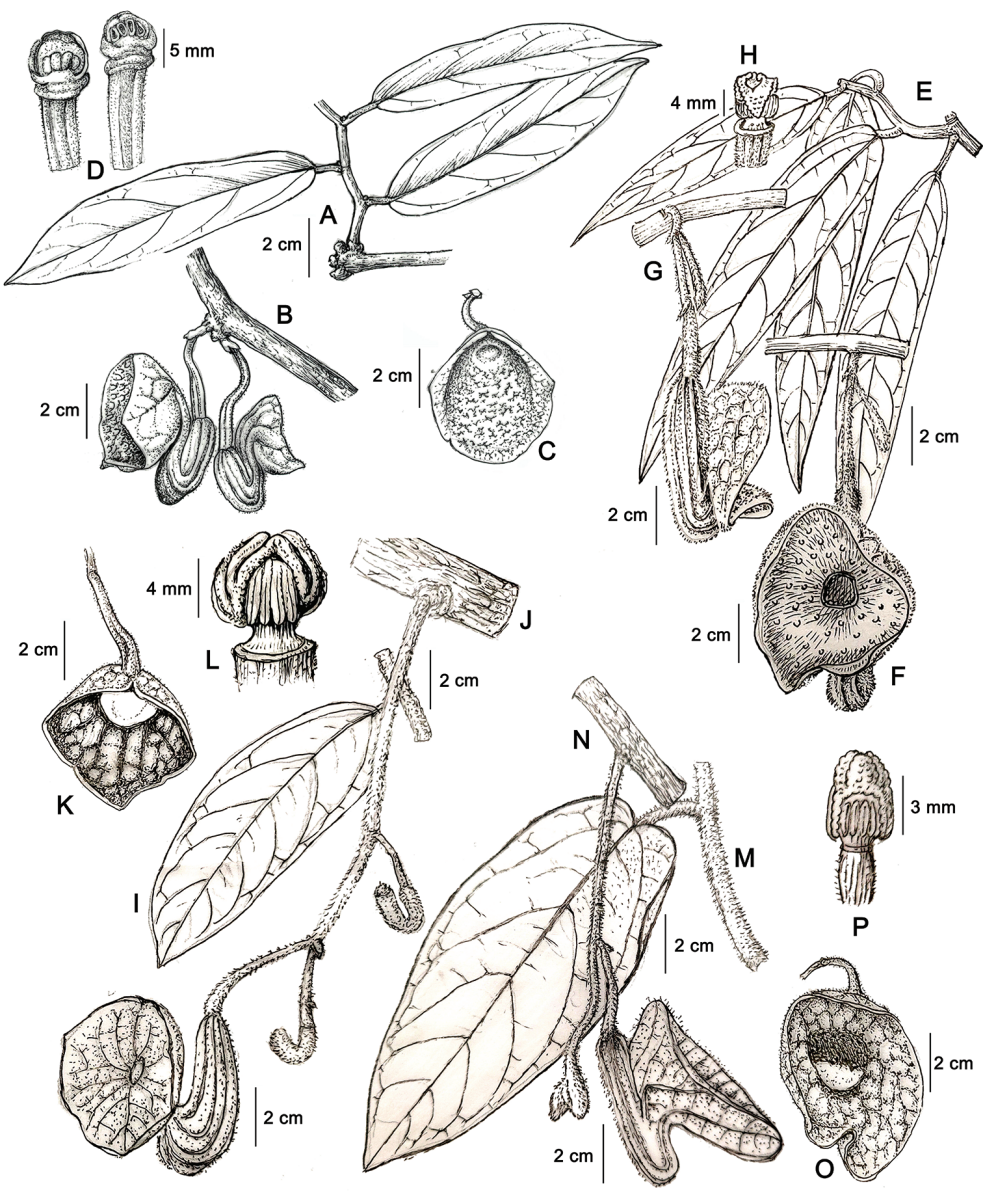
**A–D***Aristolochia yachangensis* B.G.Huang, Yan Liu & Y.S.Huang, sp. nov. **A** habitat **B** inflorescence and flowers (lateral view) **C** flower (front view) **D** anthers and gynostemium **E–H***A. championii* Merr. et Chun: **E** habitat **F** inflorescence and flower (front view) **G** flower (lateral view) **H** anthers and gynostemium **I–L***A. fangchi* Y. C. Wu ex L. D. Chow et S. M. Hwang: **I** habitat **J** inflorescence and flower (lateral view) **K** flower (front view) **L** anthers and gynostemium **M–P***A. petelotii* O. C. Schmidt: **M** habitat **N** inflorescence and flower (lateral view) **O** flower (front view) **P** anthers and gynostemium. Illustration by Wenhong Lin (based on the illustrations from Flora Reipublicae Popularis Sinicae).

Figures 1 , 2 , 3 , 4A–D

#### Diagnosis.

*Aristolochia yachangensis* is morphologically similar to *A. fangchi* Y.C.Wu ex L.D.Chow & S.M.Hwang, *A. petelotii* O.C.Schmidt and *A. championii* Merr. & Chun, but can be distinguished from them by stems irregularly striate, sparsely yellowish-brown pubescent or glabrous; leaf blade 1.5–3 cm wide; cymes on old woody stems; basal portion of perianth tube 2–3 cm long, shorter than the upper; limb yellow, with dark purple mural–like stripes; throat yellow; capsule ellipsoid. Detailed morphological comparisons amongst the four species of *A. yachangensis*, *A. championii*, *A. petelotii* and *A. fangchi* are summarised in Table 1.

**Table 1.** Morphological comparisons of key characters amongst *Aristolochia yachengensis*, *A. fangchi*, *A. petelotii* and *A. championii*.

Characters	*A. yachengensis*	*A. fangchi*	*A. petelotii*	*A. championii*
Young stem	irregularly striate, sparsely yellowish-brown pubescent or glabrous	obscurely striate, brown villous	striate, densely spreading yellowish-brown villous	striate, densely yellowish-brown villous
Leaf blade	lanceolate to elliptic–lanceolateor linear–lanceolate, 5–15 × 1.5–3 cm, base rounded or widely cuneate, lateral veins 5–8 pairs	oblong to ovate–oblong, rarely ovate–lanceolate, 6–15 × 3–5.5 cm, base rounded or cordate, lateral veins 4–6 pairs	narrowly ovate, ovate–oblong or lanceolate–ovate, 14–22.5 × 7–13 cm, base shallowly cordate, lateral veins 4–6 pairs	lanceolate to elliptic–lanceolate or linear–lanceolate, 15–30 × 2–5 cm, base rounded or shallowly cordate, lateral veins 6–15 pairs
Pedicel	1–2 cm long, densely yellowish-brown pubescent	5–7 cm long, densely brown villous	4–4.5 cm long, densely brown villous	3–4 cm long, densely brown villous
Perianth tube	basal portion of tube 2–2.5 × 0.6–1 cm, shorter than the upper part, outside of tube mauve, densely yellowish-brown pubescent	basal portion of tube 4–5 × 1–1.5 cm, longer than the upper, outside of tube purple, with white blotches or not, densely villous	basal portion of tube 5–6.5 × 1–2 cm, longer than the upper, outside of tube pale-yellow or mauve, densely villous	basal portion of tube 5–7 × ca. 1.5 cm, longer than the upper, outside of tube mauve, densely villous
Limb	yellow, with dark purple mural–like stripes	dark purple, with white blotches	dark-purple, with white stripes	dark purple
Throat	yellow	white	milk-white mixed with black	yellow, with dark purple pots
Capsule	ellipsoid, 6–10 × 2.5–3.5 cm, glabrous	cylindrical, 5–10 × 3–5 cm, villous	narrowly ellipsoid, 10–15 × 5–8 cm, yellowish-brown villous	ellipsoid, 6–8 × ca. 3 cm, villous

#### Type.

China. Guangxi Zhuang Autonomous Region: Baise City, Leye County, Huaping Town, Zhongjing (Yachang Orchid National Nature Reserve), 24°49.367'N, 106°24.029'E, 1341 m a.s.l., 29 July 2019, *Z. C. Lu et al. 20190729YC4141* (holotype: IBK!; isotypes: IBK!, GXMG!).

#### Description.

Shrubs climbing. Stems terete, irregularly striate, sparsely yellowish-brown pubescent or glabrous. Branchlets densely yellowish-brown pubescent. Leaf blade leathery, lanceolate to elliptic–lanceolate or linear–lanceolate, 5–15 × 1.5–3 cm, apex narrowly acuminate, base rounded or broadly cuneate, margin entire, adaxially glabrous except the pubescent midnerve and lateral veins, abaxially shallowly yellowish-brown pubescent, basal veins 3, lateral veins 5–8 pairs, conspicuous on both surfaces; petiole 1–1.5 cm long, slightly distorted, densely yellowish-brown pubescent. Cymes on old woody stems, 1–5–flowered; pedicel 1–2 cm long, pendulous, densely yellowish-brown pubescent; bracteole ovate–lanceolate, ca. 4 × 2 mm, densely yellowish-brown pubescent; perianth tube horseshoe–shaped; basal portion of tube 2–2.5 × 0.6–1 cm, shorter than the upper part, near the base of inner dark purple, densely villous, outside of tube mauve, densely yellowish-brown pubescent; upper portion of tube 2.5–3 × 0.5–0.8 cm, inner yellow, with dark purple stripes; limb subrotund–peltate, 4–6 cm in diam., yellow, with dark purple mural–like stripes, abaxially densely brown pubescent, margin shallowly 3–lobed, lobes apex mucronate; throat suborbicular, 0.5–1 cm in diam., yellow; anthers oblong, 2–4 × 1 mm, adnate to the gynostemium base, opposite to the lobes; ovary terete, ca. 1.5 × 0.3–0.5 cm, 6–angled, densely brown pubescent; gynostemium 3–lobed, margin glabrous and papillary. Capsule ellipsoid, 6–10 × 2.5–3.5 cm, 6–angled, glabrous.

#### Phenology.

The new species was observed flowering from March to May and fruiting from June to August.

#### Etymology.

The specific epithet is derived from the type locality, Yachang Orchid National Nature Reserve, Guangxi, China. The Chinese name is given as “雅长马兜铃”.

#### Distribution and habitat.

At present, *Aristolochia yachangensis* was found only in Yachang Orchid National Nature Reserve of north-western Guangxi, China. It grows on limestone hillside at an elevation of ca. 1340 m. The slope direction is to the southwest, the slope is up to 40°, the tree layer is up to 15 m tall, the canopy cover is 70%, the shrub layer cover is 80% and the herb layer cover is 45%. Its associated species include *Quercus variabilis* Blume (Fagaceae), *Celtis sinensis* Pers. (Ulmaceae), *Platycarya longipes* Wu (Juglandaceae), *Toxicodendron succedaneum* (L.) Kuntze (Anacardiaceae), *Yua thomsonii* (Laws.) C.L.Li (Vitaceae), Pteridium aquilinum (L.) Kuhn var. latiusculum (Desv.) Underw. ex A.Heller (Pteridiaceae), *Miscanthus sinensis* Andersson (Gramineae) etc.

#### Conservation status.

Thus far, *Aristolochia yachangensis* has been found only from the type locality. The only subpopulation is located within a protected region and has seven individuals, including two mature ones. Based on the present study, its Extent of Occurrence (EOO) is less than 100 km^2^ and the known Area of Occupancy (AOO) is less than 0.5 km^2^. According to Guidelines for Using the IUCN Red List Categories and Criteria (IUCN Standards and Petitions Committee 2019), *A. yachangensis* should be given a Vulnerable (VU) status, based on the criteria D2 of IUCN. As a newly-found species, however, it is probable that more subpopulations of *A. yachangensis* could be found in similar habitats of limestone areas of north-western Guangxi and southern Guizhou, China in the future.

#### Additional specimens examined (paratypes).

China. Guangxi Zhuang Autonomous Region: Baise City, Leye County, Huaping Town, Zhongjing (Yachang Orchid National Nature Reserve), 24°49.367'N, 106°24.029'E, 1341 m a.s.l., 21 April 2019, *Y. J. Luo & S. W. Li 20190421001* (IBK); the same location, 17 May 2019, *Y. J. Luo et al. YC4439* (IBK).

## Discussion

*Aristolochia yachangensis* is unique in morphology. It is mostly similar to *A. fangchi*, *A. petelotii* and *A. championii*, but can be distinguished from all other *Aristolochia* species mainly based on the morphological characters of inflorescence, perianth tube, limb and throat. *A. yachangensis* can be further distinguished from morphologically-close species with the following key.

## Key to *Aristolochia yachangensis* and morphologically-close species

**Table:** 

1	Limb adaxially papillate or upper papillate, lower smooth	**2**
–	Limb adaxially smooth	**3**
2	Basal portion of tube shorter than the upper; limb adaxially yellow, with dark purple stripes	**4**
–	Basal portion of tube longer than the upper; limb adaxially dark purple	***A. championii***
3	Limb adaxially yellow	**5**
–	Limb adaxially dark purple or reddish-purple, sometimes with yellow or white blotches	**6**
4	Leaf blade narrowly ovate to ovate–oblong, base cordate; petiole 4–5 cm long; limb 3–4 cm in diam	***A. huanjiangensis***
–	Leaf blade lanceolate to elliptic–lanceolate or linear–lanceolate, base rounded or broadly cuneate; petiole 1–1.5 cm long; limb 4–6 cm in diam	***A. yachangensis***
5	Leaf blade ovate to narrowly ovate; limb ca. 2.5 cm in diam.; lobes of gynostemium pubescent	***A. pilosistyla***
–	Leaf blade oblanceolate to lanceolate–elliptic; limb 4–6 cm in diam.; lobes of gynostemium glabrous	***A. versicolor***
6	Limb small, ca. 3 cm × 1.5–2 cm, adaxially without blotches	***A. fulvicoma***
–	Limb large, 4–13 cm in diam	**7**
7	Leaf blade lanceolate–oblong or narrowly oblong, base narrowly auriculate, lateral veins 8–12; limb 8–13 cm in diam	***A. westlandii***
–	Leaf blade ovate, oblong or ovate-oblong, rarely ovate-lanceolate, base cordate or rounded; limb no more than 8 cm in diam	**8**
8	Leaf blade base rounded, rarely cordate; limb dark purple, with pale yellowish blotches	***A. fangchi***
–	Leaf blade base cordate; limb dark purple or reddish-purple, with white blotches or pale yellowish, without blotches	***A. petelotii***

## Supplementary Material

XML Treatment for Aristolochia yachangensis
